# Proof of Concept for the Detection of Local Pressure Marks in Prosthesis Sockets Using Structural Dynamics Measurement

**DOI:** 10.3390/s21113821

**Published:** 2021-05-31

**Authors:** Constanze Neupetsch, Eric Hensel, Burkhard Kranz, Welf-Guntram Drossel, Thomas Felderhoff, Christoph-Eckhard Heyde

**Affiliations:** 1 Fraunhofer Institute for Machine Tools and Forming Technology, 09126 Chemnitz, Germany; eric.hensel@iwu.fraunhofer.de (E.H.); burkhard.kranz@iwu.fraunhofer.de (B.K.); welf-guntram.drossel@iwu.fraunhofer.de (W.-G.D.); 2 Professorship Adaptronics and Lightweight Design, Faculty of Mechanical Engineering, Chemnitz University of Technology, 09111 Chemnitz, Germany; 3 Department of Orthopaedic, Trauma and Plastic Surgery, University of Leipzig Medical Center, 04103 Leipzig, Germany; Christoph-Eckhard.Heyde@medizin.uni-leipzig.de; 4 Faculty of Information Technology, Fachhochschule Dortmund-University of Applied Sciences and Arts, 44139 Dortmund, Germany; felderhoff@fh-dortmund.de

**Keywords:** residual limb, volume fluctuation, prosthesis fit, local pressure mark, structural dynamics, frequency response analysis

## Abstract

The wear comfort of a prosthesis is of great importance for amputee patients. The wear comfort can be affected by changes in the interface between the residual limb and prosthesis socket, which can be caused by time-dependent volume fluctuations of the tissue, leading to unwanted local pressure marks. The basis to ensure time-independent wear comfort of a prosthesis is to identify these changes. Common techniques for identifying these variations have a negative impact on the sensitive interface between the residual limb and prosthesis. The following paper contains a proof of concept for the detection of local pressure marks without affecting the described interface using structural dynamics measurements, exemplarily shown at a prosthetic socket for transfemoral amputees in a test bench scenario. The dynamical behaviour of the investigated system is analysed in the form of frequency response functions acquired for different pressure locations and preloads using an impact hammer for excitation and a triaxial acceleration sensor. The frequency response functions show major changes for the various boundary conditions with respect to their frequency-dependent compositions. The results demonstrate how the utilised method enables the identification of changes in local pressure marks regarding the variation of position and magnitude.

## 1. Introduction

### 1.1. Problem Description and State of the Art

The incidence of lower limb amputations is increasing worldwide due to the high rate of vascular-related diseases and traumatic accidents [1]. This trend can also be observed in Germany. Between 2011 and 2015, 52,304 amputations were carried out at the level of the hip joint or femur. This represents a share of 19.1% of all required amputations. Only in the area of the toes were more amputations performed in the period under review [2]. Amputees often use a prosthesis as a rehabilitation device to restore their appearance and ability to carry out daily activities. The prosthesis consists of several essential components. The socket provides the coupling between the residual limb and the remaining components of the prosthetic device. The soft tissues of the residual limb amputees are subject to large volume fluctuations over the course of the day. The negative influences on the tissue and on the patient’s carrying comfort result from the volume fluctuations. Fluctuations in the volume of the residual limb can lead to local pressure marks. Various studies show a heterogeneous pressure distribution and investigate the relationship between comfort and the localisation of the peak load between the residual limb and the socket [3,4,5]. In addition, other studies determine critical threshold values and pain thresholds in connection with too much strain on different areas of the residual limb [6,7,8]. Up to 50% of transtibial amputation (amputation across or involving the shinbone) patients do not use their prosthesis regularly, mainly due to low comfort caused by socket problems [9,10,11]. For transfemoral amputation (amputation across or involving the thighbone) patients, the non-use rate is even higher [12].

Orthopaedic technicians consider biomechanical parameters, but patients with lower limb amputations remain dissatisfied with prostheses [13,14]. The sense of comfort primarily refers to the pressure relationships between the socket and residual limb. The socket fit, the type of prosthesis suspension and the alignment of the prosthesis can change the pressure on the residual limb. Excessive and/or long-term interfacial stresses lead to tissue degradation [15]. The evaluation of comfort corresponds to the subjective perception of the patient [16,17,18,19].

Research shows that a tissue-specific load distribution reduces the risk of lasting damage to the tissues and the residual limb. The basis for this is an adequate and measurement-based analysis of the fit of the prosthetic socket. Early measurements of interface stress were limited to a few specific areas of the residual limb. In addition, holes in the socket or liner were required to accommodate the pressure sensors. There were also problems with sensor movement, crosstalk between sensors, and interference with the interaction of the residual limb and the socket [20,21].

To address some of the challenges, other approaches have been explored. Through a neural network, for example, strain data of the socket surface should allow prediction. However, there are limitations due to the need to retrain the neural network after each socket modification [22,23]. Another approach presented in [24] pursues the idea of using an additive manufactured sensor to evaluate the interface stress on the prosthesis socket in the clinical environment. Amputees could be warned at home of excessive stress at the interface between residual limb and socket. Degradation of the residual limb tissue should thus be prevented at an early stage. The sensor must be applied to the prosthetic socket and residual limb interface for effective data analysis. Negative influences could arise in particular from the sensor thickness of 4 mm, even though this is still comparatively small. The study [25] aims to investigate the capability of fibre bragg grating (FBG)-based sensors to measure the interface pressure of the stump and socket of amputees. FBG elements were recoated with and embedded in a layer of epoxy material to form a sensing pad, which was in turn embedded in a silicone polymer material to form a pressure sensor. The integration volume required in this process is not insignificant. In addition, there are other approaches that are also limited by the bulky sensors used [6], the need for holes in the socket [25,26] or cables [27,28,29], which disrupts the environment within the socket and compromises the value of the measurements.

The shown weaknesses and the potential for optimisation in the analysis of local pressure marks in prosthesis sockets show the need for further research activities in this field. Structural dynamics measurement methods as a field of acoustics represent a promising approach for a feedback-free analysis. The transferability of experimental acoustic analysis to medical engineering problems has already been demonstrated in various studies [30,31,32,33,34,35], with particular focus in [36] on the special features of the application of structural dynamics analyses to biological specimens. Most of the application of acoustic measurement methods to biological specimens follows the objective of parameter identification, e.g., the acquisition of modal parameters and a subsequent adaptation of simulation models to increase the prediction accuracy [37,38,39,40]. Both results and basic structural dynamics considerations show that in all investigations based on structure-borne sound the prevailing boundary conditions (BCs) are essential [41]. However, investigations with structures made of carbon fibre-reinforced plastic following the geometric model of prosthesis sockets for a system characterisation with different BCs using structure-borne sound measurements are not known in the literature.

### 1.2. Used Approach

**Figure 1:**
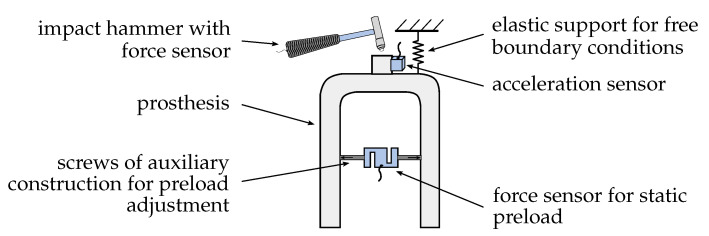
Representation of the used test setup with sensor locations, support and auxiliary construction.

The analyses presented below deal with the capability to identify changes at the interface between the prosthesis socket and residual limb using structural dynamics measurements. The objective was to detect these changes with a minimal set of sensors to reduce the effort for a final implementation of a smart prosthesis. In Figure 1, a schematic representation of the used approach to reach the mentioned objective is shown.

As can be seen, the changes at the interface between prosthesis socket and residual limb were imitated using an auxiliary construction consisting of a force sensor and two screws. The force sensor of the auxiliary construction was utilised to obtain the static preload which could be adjusted with the two screws by revolving them against each other. This auxiliary construction could be moved to other positions to change the location of local pressure marks. Hence, the auxiliary construction was used to represent the force that is applied on the prosthesis socket by the residual limb. For structural dynamics measurements, the prosthesis was supported with an elastic support to realise idealised BC. A more detailed description of the chosen BC is given in Section 3.1. Finally, the dynamical behaviour of the prosthesis was evaluated using an impact hammer for excitation and an acceleration sensor to acquire the corresponding system’s response. Excitation and response were calculated in the form of frequency response functions (FRFs), which were utilised to investigate changes in magnitude and position of preloads applied using the described auxiliary construction.

## 2. Object of Investigation

**Figure 2:**
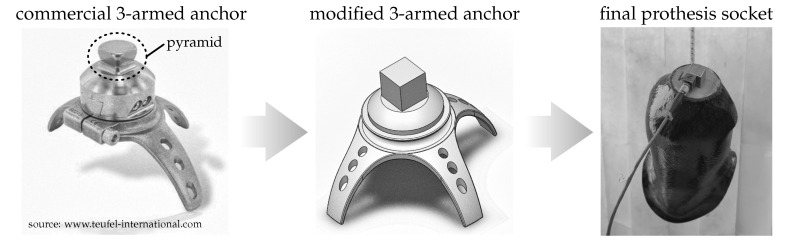
Modification of the 3-armed anchor and integration into final prosthesis socket.

The test object was an anatomically shaped prosthesis socket that was manufactured according to the state of the art. The socket was extended by an individualised and additive manufactured anchor to realise excitation and measurement in different directions. The basic geometric shape of the anchor was derived from a commercially available three-armed anchor (cf. Figure 2). The arms were used to fix the anchor in the carbon fibre mesh. The so-called pyramid of the anchor was used to align the angle between the socket and the fitting parts of the leg and was positioned orthogonally to the force direction due to body weight. For the test execution, the design of the pyramid was changed to a cube with 15 mm edge length.

The modified anchor was manufactured from Titanium (Ti6Al4V) using 25 μm thin layers on a laser beam melting machine (Concept Laser GmbH, Lichtenfels, Germany: *type* Concept Laser M2 Cusing single laser 400 W). A scan speed up to 1200 mm/s and laser power up to 200 W were used for the laser beam melting process. The prosthesis socket was modelled with the aid of a carbon fibre composite. The basis was a patient-specific positive model of a transfemoral amputee made of plaster. After application of an underlay film, which was used as an insulating layer, several carbon fibre meshes were applied and fixed with a spray adhesive. To increase stability, a higher quantity of carbon fibre meshes were applied at positions where a large force impact is to be expected (e.g., ischial region, distal end, anchor attachment). In an intermediate step, the modified anchor was inserted into the carbon fibre mesh. For the final production of the fibre-reinforced prosthesis socket, the individual carbon fibre meshes were bonded in a consecutive production step by means of a casting resin, also referred to as matrix. A vacuum was used to create a bond between the resin and the reinforcing fibres. After this step, excess resin was removed and the socket was ground to its final shape. Therefore, the investigated prosthesis socket represented a state of the art model with a modified anchor.

## 3. Test Setup and Data Acquisition

### 3.1. Test Setup

The following section contains the description of the used test setup. In order to minimise environmental influences, the acquisition of experimental data was carried out under idealised BCs. In terms of structural dynamics measurements, idealised BCs can be realised either in a free or fixed (also known as grounded) configuration. The choice of the BC depends on the analysed structure. If a fixed support is chosen, one has to ensure the rigidity of the ground, which means that the test object must not be influenced by the dynamical behaviour of the structure where it is mounted. In most cases, and especially for smaller structures such as the investigated prosthesis socket, a free support can be realised in a more straightforward way by using soft springs or elastic bands. The use of freely supported conditions leads to the presence of rigid body modes (RBMs) depending on the mass and inertia properties of the tested structure. For an ideal free support, the RBM occurs at 0 Hz. In praxis, such an ideal free BC cannot be realised since the connection to the ground, for example, via soft springs, has a defined stiffness shifting the RBM to frequencies >0 Hz. Thus, the free support in experimental analyses is chosen in a way that the RBMs are less than 10% to 20% of the frequency of the first flexural mode [42], p. 90.

**Figure 3:**
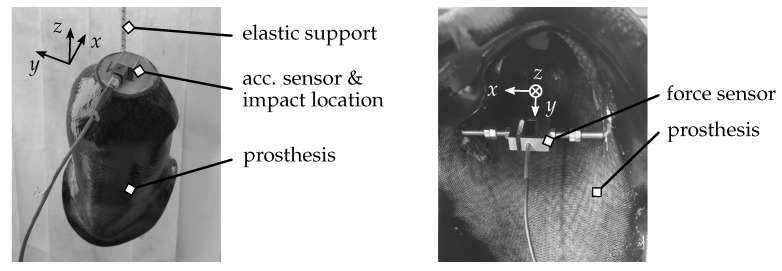
Test setup for experimental analysis of the prosthesis socket. (**left**) Outer view of the test setup showing prosthesis, elastic support and location of excitation and response of the system. (**right**) Inner view showing the position of the force element.

The test setup for the investigated prosthesis socket in a freely supported configuration is shown in Figure 3.

The left part of Figure 3 contains the elastic support realised by an elastomer strap as well as the location where the system’s dynamical behaviour is analysed. The hole in the prosthesis socket provided for the ejection valve was used to apply the elastic support. Within the presented investigations, the response of the prosthesis socket was acquired at the same location where it was excited. The response of the system was obtained by a triaxial acceleration sensor (PCB Piezotronics, Inc., Depew, NY, USA: *type* 356A45). On the other hand, the excitation was realised using an impact hammer with a mass of 160 g (PCB Piezotronics, Inc.: *type* 086C03). Due to the use of a hammer as the excitation source, the system was excited by an impulse which basically depends on the hammer mass and the applied tip. The use of different tips enables the excitation of various frequency ranges. Softer tips are usually used for the lower frequency range since the impulse has a broader shape in time domain due to the elastic deformation of the tip. On the other hand, a hard tip is used to excite the investigated system at higher frequencies. In the present case, a plastic tip was used, which is a good compromise between the lower and higher frequency ranges. The choice of the hammer tip was based on the frequency range where differences in inertances were obtained (cf. Section 4.2 and Section 4.3). The resulting excitation spectrum, as well as other measurement indicators, is shown and discussed in Section 4.1. For data acquisition, a multi-channel measurement system (Müller-BBM VibroAkustik Systeme GmbH, Planegg, Germany: *type* PAK MKII) was used. The data acquisition and the corresponding settings used within the measurements are part of the following Section 3.2.

The right part of Figure 3 shows an inner view of the prosthesis socket. As can be seen, an auxiliary construction containing an additional force sensor was applied. Due to the design of the auxiliary construction using two screws on each side, different preloads could be realised for the subsequent dynamical measurements. The force sensor was used to obtain the current preload imitating various pressures at different locations of the prosthesis socket.

### 3.2. Data Acquisition

As described in the previous section, the measurements were carried out at a single position where excitation and response location coincided. The evaluation of the system’s dynamics at this location was based on the obtained inertances, also referred to as accelerances. The inertances *A* were calculated as the ratio of acceleration *a* and force *f*

(1) $$A=\frac{a}{F}$$

obtained in form of a FRF. For structural dynamics measurements, different aspects need to be considered when using and calculating FRF to describe the system’s behaviour. In praxis, noise, for example, electrical or mechanical noise, will be present in nearly every situation. In order to minimise these noise effects, one has to perform an averaging of repeating measurements which means that the data for a single impact location are acquired and averaged. The number of averages depends on the given structure. The present investigations were carried out using five averages for each excitation degree of freedom (DoF).

**Figure 4:**
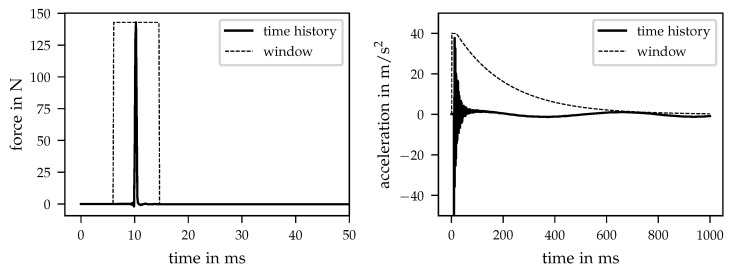
Applied window functions and example time histories. (**left**) Time history and window function for excitation/force signal. (**right**) Time history and window function for response/acceleration signal.

Another important issue is the application of window functions to the acquired time signals. A common problem by performing impact measurements is the presence of leakage, which is caused by the transformation from the time to frequency domain (using Fast Fourier Transform). Since the signals are repeated periodically, mismatches between start and end points lead to an effect that is not based on the behaviour of the investigated structure but on signal processing. Hence, the leakage effect should be minimised. In praxis, the reduction in leakage is realised by the application of window functions to the acquired signals. In case of impact measurements, different windows are used for excitation and response. The two windows applied within the underlying measurements are shown in Figure 4. In addition, the diagrams contain two example time histories of excitation (force) and response (acceleration).

The left part of Figure 4 shows an example time history of the force and the corresponding window function. For impact hammer measurements, usually a rectangular window is applied that is centred around the maximum of the acquired force. In addition to the minimisation of leakage as mentioned above, the applied rectangular window reduces the existing noise when the impact faded down to zero. In contrast, the window function of the response signal holds an exponential shape that can be seen in the right part of Figure 4. In the same way as the window of the excitation signal, the exponential window forces the acceleration signal nearly down to zero to avoid leakage and to reduce remaining noise. Figure 4 clarifies the effect of the exponential window function since the RBMs do not fall down to zero within the measured time blocks. The data acquisition was carried out using time blocks of 1 s, a resulting frequency resolution of 1 Hz and a frequency range up to 3200 Hz with a corresponding sampling frequency of 8192 Hz.

The different FRF were obtained for different preloads where the static forces were kept nearly constant during impact hammer measurements. The system was excited in three different DoFs using the cube at the distal end of the prosthesis socket (cf. Figure 3). To evaluate the changes within the obtained data due to a change in preload position, two different locations of the auxiliary construction were investigated. The right part of Figure 3 shows position 1. The auxiliary construction was rotated about 90° about the *z* axis for the second position.

## 4. Results

The following section is split into three different parts. At first, a single measurement is shown and the content focuses on the evaluation of measurement criteria such as the valid frequency range. The second subsection deals with the resulting FRF and their changes due to preload variation. Finally, a statistical analysis of the obtained results is shown.

### 4.1. Preliminary Considerations

**Figure 5:**
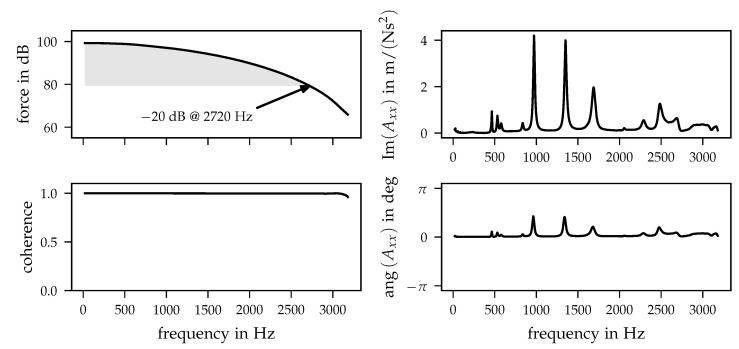
Measurement results of a single example position with a preload of 51 N. (**left**) Force spectrum (dB reference *F*_0_ = 1·10^−6^ N) and coherence function. (**right**) Imaginary part and phase of inertance.

Before investigating the influence of various preloads on the system’s dynamical behaviour, a brief evaluation of impact hammer measurement quality is given. Figure 5 contains different frequency-dependent quantities of a single measurement with averaged values of five repeating impacts (cf. Section 3.2).

The upper left diagram shows the force excitation spectrum. For impact measurements, a rule of thumb is that the excitation is valid for a decrease in force by −20 dB in comparison to the low frequency range. In addition, the frequency at 2720 Hz is marked where the force spectrum hits the limit of −20 dB. Hence, by only analysing the −20 dB decrease, the measurement is valid for the frequency range up to 2720 Hz. The described −20 dB decrease is just a first parameter when investigating the valid frequency range of impact hammer measurements. The second quantity is the coherence function, which was used for investigating the quality of obtained data (cf. [42], pp. 130–132). The coherence function offers information if excitation and response signals (in terms of impact hammer measurements) are related to each other. For an ideal measurement, the coherence is equal to unity where, in praxis, it should be greater than 0.8. For example, the coherence can be used to identify frequency ranges with dominating noise due to insufficient system excitation. In addition, it indicates the presence of interfering signals, e.g., if the vibration at a certain frequency is caused by another excitation source than the impact hammer. An example coherence obtained within the prosthesis socket measurements is shown in the lower left diagram of Figure 5. As can be seen, the coherence has no major drops within the entire considered frequency range up to 3200 Hz.

After analysing the different measurement quality indicators described above, the resulting FRF should be discussed in detail. In the right part of Figure 5, the imaginary part of an example FRF is shown in the upper diagram and the lower plot contains the corresponding phase. The evaluation of the imaginary part of inertance FRF was chosen since it is related to mode shapes of the system (cf. [43], p. 612). Regarding the quality and correctness of the measurement, one has to focus on the sign of both curves, which both point in positive directions. To evaluate this in terms of the measurement, a closer look needs to be taken on the type of FRF printed above. The FRF in Figure 5 is a driving point FRF, which means that the excitation DoF and response DoF coincide. In case of the FRF in Figure 5, the DoF in the transverse direction with respect to the longitudinal axis of the prosthesis socket is shown (cf. Figure 3, *x* direction). A driving point FRF is a special type of FRF and has to meet different requirements. As mentioned above, the obtained inertances represent the ratio of acceleration and response. Force and acceleration need to be in phase because the location is the same. In praxis, this cannot be ensured by using an impact hammer and acceleration sensors since they can only be nearly at the same location physically. If the phase changes due to a relative motion between force and acceleration, the assumption of a driving point loses its validity. In consequence of the in phase behaviour of force and acceleration, all peaks of the imaginary part of the inertance FRF must point in the same direction.

It can be stated that the acquired FRF meets the described conditions over the entire frequency range, which is clarified by Figure 5, and thus it can be stated that the dynamical properties were acquired in a sufficient way. Within the following evaluation of measurements, two different driving point FRFs are used to analyse the change in the system’s dynamical behaviour due to the change in preload quantity and position.

### 4.2. Influence of Preloads on Obtained Inertances

This section contains the experimental results in dependency of various preloads. The data were acquired for three different preloads of approx. 20 N, 40 N and 50 N. In addition, the auxiliary construction for realising the static preload of the system was applied at two different locations. For position 1, the preload coincided with the global *x* axis (cf. Figure 3). In comparison, the auxiliary construction mounted in position 2 pointed in the global *y* direction.

**Figure 6:**
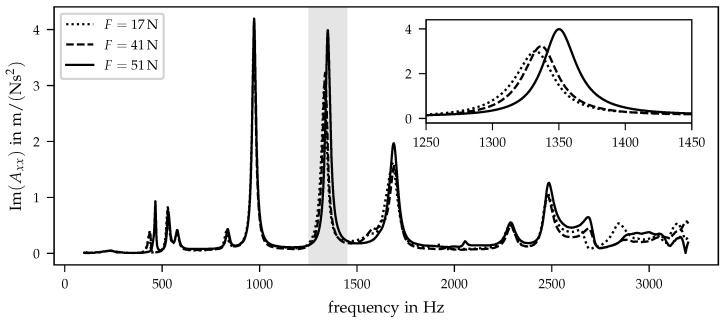
Imaginary part of inertance $A_{xx}$ for three different preloads and the auxiliary construction in position 1. The small diagram in the right corner shows the inertances in the frequency range from 1250 Hz to 1450 Hz.

Figure 6 shows the imaginary part of inertance $A_{xx}$ where excitation and response point in the global *x* direction and the auxiliary construction is in position 1 (cf. Figure 3).

The three curves obtained for different preloads show similar frequency-dependent behaviours. Especially in the frequency range from 700 Hz to 1800 Hz, three dominant peaks can be detected that were caused by natural frequencies of the investigated system. The corresponding mode shapes cannot be analysed with the current test setup since the data are only acquired at one location. For a detailed mode shape analysis, a higher spatial resolution is required. As mentioned above, the measurements shown here focus on the feasibility of the detection of local pressure changes in the prosthesis socket’s interface. In terms of final implementability, a minimal number of sensors is required due to reduction in costs and complexity. Therefore, this first analysis was carried out using just one sensor location knowing that corresponding mode shapes cannot be evaluated. The mentioned peaks in Figure 6 show different behaviours regarding their dependency on various preloads. For example, the first peak at approx. 1000 Hz does not show any dependency on preloads. In contrast, the second peak within the frequency range from 700 Hz to 1800 Hz is shifted to higher frequencies with increasing preloads. A more detailed view of this second peak is shown in the right corner of Figure 6. The detail view clarifies that the maximum additionally moves to higher values with increasing preloads.

**Figure 7:**
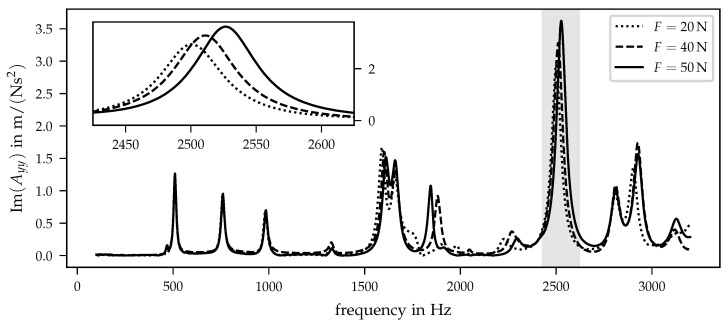
Imaginary part of inertance $A_{yy}$ for three different preloads and the auxiliary construction in position 2. The small diagram in the left corner shows the inertances in the frequency range from 2425 Hz to 2625 Hz.

Figure 7 contains the inertances in the *y* direction and the auxiliary construction applied in position 2.

The shape of the FRF differ in comparison to the inertances in *x* direction, which is plausible due to the non-symmetrical form of the investigated prosthesis socket. The differences between both directions, *x* and *y*, are discussed in detail in the following Section 4.3. In Figure 7, one dominant peak within the considered frequency range can be localised at approx. 2500 Hz. Again, the diagram is supplemented by a detailed view of the frequency range of interest. Note, that the preloads slightly differ in comparison to the preloads at position 1 due to the sensitivity of adjusting the used auxiliary adapter with the two screws. The peak at approx. 2500 Hz shows a similar dependency from preloads such as the inertance in the *x* direction at position 1. First of all, increasing preloads leads to a frequency shift to higher values. In addition, the imaginary parts of inertance increase for larger preload values. Before analysing the inertances’ preload dependency, a brief comparison between the two investigated preload positions will be given in the following section.

### 4.3. Influence of Preload Position

The previous part contains the inertances of the two driving points in global *x* and *y* directions with dependency on three preload steps, where $A_{xx}$ and $A_{yy}$ were evaluated for different preload positions. As mentioned above, position 1 is shown in Figure 3 and position 2 was realised by rotating the auxiliary construction about 90° around the global *z* axis. Within this section, the differences between these two positions are discussed.

**Figure 8:**
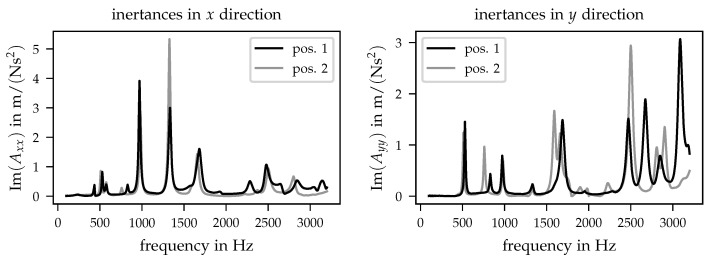
Comparison of driving point inertances for different preload positions and preload level at approx. 50 N. (**left**) Imaginary part of input inertances $A_{xx}$. (**right**) Imaginary part of input inertances $A_{yy}$.

The driving point inertances $A_{xx}$ and $A_{yy}$ for both positions are compared in Figure 8. For the preload level, the highest static force of approx. 50 N was chosen for representation.

In the left diagram of Figure 8, the inertances show only minor changes with respect to the corresponding frequencies where the peaks can be detected. With exception of the peak at 1300 Hz, the imaginary parts show comparable values between both positions for the entire frequency range considered within the present investigations. In comparison, the inertances $A_{yy}$ (cf. Figure 8, right diagram) differ in terms of imaginary part and frequency. The differences between both diagrams and the shown dependencies can be explained by the sensitivities of the corresponding mode shapes on the applied preload positions. Hence, the inertance in the *x* direction can be less sensitive to a change in preload positions. Note that this conclusion can only be drawn for the two investigated positions. Other preload positions may cause a more relevant influence on the driving point inertance in the *x* direction but were not included in the current measurement data base. Further aspects will be discussed in Section 5.

### 4.4. Dependency on Preload

**Figure 9:**
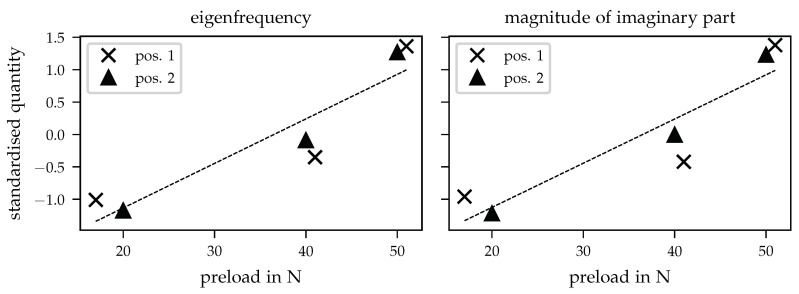
Standardised quantities dependency on preload for both investigated preload positions. The dashed line indicates the corresponding linear regression. (**left**) Standardised eigenfrequencies. (**right**) Standardised peaks of imaginary part of acquired inertances.

In this section, the relations between the acquired inertances and the preloads will be presented. The dependencies will be evaluated separately for the eigenfrequencies and for the corresponding imaginary parts of the inertances. Before discussing the results, a brief description of the calculation of resulting values should be given. Section 4.2 contains the driving point inertances in *x* and *y* direction where the detail views in the corresponding figures show the frequency ranges with a similar dependency of the FRF on the different preloads (cf. Figure 6 and Figure 7). In order to gain a better comparability of the different quantities, a standardisation

(2) $$z=\frac{x-\mu }{\sigma }$$

was carried out for both properties, the eigenfrequencies and the chosen peaks of imaginary parts. In Equation (2), *x* represents the raw data (e.g., eigenfrequencies) with the corresponding mean value μ and standard deviation σ and *z* referring to the resulting standardised data. The standardisation was applied to each single data set containing the eigenfrequencies and peaks of imaginary parts for the three preload steps obtained for both positions. For the given problem, a standardisation is plausible since the identified changes occur at different frequencies for various positions and directions, respectively. The corresponding results of standardised values of eigenfrequencies and peaks of imaginary parts in dependency on the static preload are shown in Figure 9. Within the further descriptions below, the explicit characterisation “standardised” is omitted.

First of all, one can observe that both, eigenfrequencies and peaks of imaginary parts, rise with increasing preloads, which is pointed out by the additional dashed line representing a linear regression function. Here, it should be noted that the linear regression is inserted due to the better comparability of the single values. Based on the current data sets, a final regression analysis does not seem useful and needs an enhanced database that should be able to undergo further experimental investigations. The change in eigenfrequencies can be explained by a local stiffening of the prosthesis socket since the mass of the system, consisting of prosthesis and auxiliary construction, did not vary over all measurements. The right part of Figure 9 contains the dependency of the inertances’ imaginary part peaks increasing for higher preloads. Higher values of FRF imaginary parts usually indicate the presence of a lower damping at the observed frequency. Based on the current available data, the changes in imaginary parts cannot be explained by decreasing damping since the damping ratio calculated using 3 dB bandwidth does not change in the expected way. To clarify this behaviour, additional investigations with focus on damping parameter evaluation are necessary.

## 5. Discussion

The goal of the presented investigation was to verify whether structural dynamics measurements can be used to identify changes in the interface between prosthesis sockets and residual limb. This question can be separated into two different parts. At first, the experimental data should provide the opportunity to represent changes in local pressure marks. The results presented in the previous section indicated that a variation of preloads can be detected by changes in the corresponding FRF where shifts in eigenfrequencies as well as in imaginary parts of inertances were recognised. On the other hand, the obtained data should enable the determination of different pressure positions that can change during the day when the prosthesis is worn. As described in Section 4.3, the acquired inertances show major differences between the two investigated preload positions. Based on the obtained data, the changes in the preload’s position and value can be observed.

The presented investigations constitute only a first set of analyses and should be enhanced to clarify the following aspects. In the performed experiments, only one sensor position has been used to obtain the driving point FRF. The driving point inertance in the *y* direction (cf. Figure 8) show major changes between both realised positions, and thus it seems to be a promising candidate for the identification of different pressure locations using a minimal number of sensors. Based on the current data set, it is not possible to draw general conclusions regarding the observability of all possible pressure locations with one accelerometer. In addition, the spatial resolution of identified pressure locations needs to be analysed in detail. Another aspect is the shown dependency between the applied static preload forces and changes in eigenfrequencies and imaginary parts of FRF as well. Additional load steps may be used to clarify the relation between the shown quantities and if a linear model is sufficient or a non-linear approach is required. In addition, the transferability of results obtained for a simplified test setup used within the present investigations to the interface between prosthesis sockets and residual limb needs to be analysed.

The discussed aspects should be part of further research works but the presented results clarify the capability of using structural dynamics methods for identifying changes in prosthesis sockets and residual limb interfaces. In further work, the evaluation of the system excitation and response detection to be defined (e.g., by means of impulse hammer and acceleration sensor as in the presented study) should be further investigated, first with regard to a clear identification of local pressure marks. In addition, the optimal number of required sensor measurement points must be determined. The results presented here suggest that the method is suitable for realising a spatially resolved detection of local pressure points with a small number of sensor measuring points. This results in an enormous potential with regard to the complexity of the measurement technology to be integrated, and thus the required weight. As a first step, these investigations should be carried out in laboratory tests using realistic synthetic materials in order to simulate the volume fluctuations of human tissue. Test series in which the targeted boundary conditions can be changed would provide a sufficient database for the subsequent evaluation of measurement data. By analysing characteristics and patterns, a feedback-free real-time analysis of a prosthesis socket can be implemented in the future. The transferability of the findings must be validated with different users and conventional measurement technology. Subsequently, further optimisations are conceivable. As an example, the exploitation of the tread as a system excitation can be mentioned, which allows a reduction in the complexity of the measurement technique necessary for the determination of the fit.

The results show that the identification of local pressure marks is realisable with the presented approach (cf. Figure 6, Figure 7 and Figure 8). It should be stated here that the detection of the described changes is based on the comparison to a reference or initial (inertance) function. This initial state needs to be known and can be obtained, for example, during the initial daily fitting of the prosthesis.

## 6. Conclusions

Current care of amputee patients relies mostly on the expertise of orthopaedic technicians and the clinical assessment of physicians, as well as subjective feedback from patients. Monitoring and sensor technologies have the potential to complement current clinical practice by improving therapeutic, diagnostic, and prognostic outcomes. Instrumented sockets, with appropriate feedback-free sensing, could be used to collect field data on socket and limb variables in the future. For smart prostheses that specifically change their shapes, adequate and measurement-based analysis of the fit is the basis for adapting the socket system.

The presented investigation shows that a system characterisation by means of acoustic measurement methods on structures made of carbon fibre-reinforced plastic according to the geometric model of prosthesis sockets is possible in general. Compared to other methods, no changes to the topology of the prosthesis socket are required. The sensitive interface between the residual limb and the prosthesis also remains unaffected, thus ruling out any negative effect of the measurement technique on the tissue in this area.

## Data Availability

The data presented in this study are available on request from the corresponding author.

## Sample Availability

Samples of the modified prosthesis stock are available from the corresponding author.

## Abbreviations

The following abbreviations are used in this manuscript:

BC	Boundary condition
DoF	Degree of freedom
FBG	Fibre bragg grating
FRF	Frequency response function
RBM	Rigid body modes
